# B cell depletion after treatment with rituximab predicts relapse of IgG4-related disease

**DOI:** 10.1093/rheumatology/keae248

**Published:** 2024-05-23

**Authors:** Marco Lanzillotta, Giuseppe Alvise Ramirez, Raffaella Milani, Lorenzo Dagna, Emanuel Della-Torre

**Affiliations:** Università Vita-Salute San Raffaele, IRCCS San Raffaele Scientific Institute, Milan, Italy; Unit of Immunology, Rheumatology, Allergy and Rare Diseases (UnIRAR), IRCCS San Raffaele Scientific Institute, Milan, Italy; Università Vita-Salute San Raffaele, IRCCS San Raffaele Scientific Institute, Milan, Italy; Unit of Immunology, Rheumatology, Allergy and Rare Diseases (UnIRAR), IRCCS San Raffaele Scientific Institute, Milan, Italy; Unit of Immunohematology and Transfusion Medicine, IRCCS San Raffaele Scientific Institute, Milan, Italy; Università Vita-Salute San Raffaele, IRCCS San Raffaele Scientific Institute, Milan, Italy; Unit of Immunology, Rheumatology, Allergy and Rare Diseases (UnIRAR), IRCCS San Raffaele Scientific Institute, Milan, Italy; Università Vita-Salute San Raffaele, IRCCS San Raffaele Scientific Institute, Milan, Italy; Unit of Immunology, Rheumatology, Allergy and Rare Diseases (UnIRAR), IRCCS San Raffaele Scientific Institute, Milan, Italy

**Keywords:** IgG4-related disease, rituximab, B cells, plasmablast, biomarker, remission, maintenance treatment

## Abstract

**Objectives:**

B cell depletion therapy with rituximab is effective in most patients with IgG4-related disease (IgG4-RD) but requires repeated cycles to prevent disease flares. We here aimed to assess B cells after rituximab to predict relapse of IgG4-RD and guide retreatment.

**Methods:**

Patients with active IgG4-RD included in this retrospective study fulfilled the ACR/EULAR Classification Criteria. Total CD19^+^ B cells, plasmablasts, naïve and memory B cells were measured on peripheral blood by flow-cytometry at baseline and 6 months after rituximab. All patients were treated with two 1 g infusions of rituximab 15 days apart and monitored for 48 months. Disease response was assessed using the IgG4-RD Responder Index.

**Results:**

Thirty-three patients were included. Six months after rituximab, disease response was observed in all patients. Complete depletion of CD19^+^ B cells, plasmablasts, naïve and memory B cell depletion was achieved in 30%, 55%, 39% and 42% of cases, respectively. Twenty-three relapses (70%) were observed at a median time of 24 months after rituximab. Relapse rate was significantly higher in patients who failed to achieve complete depletion of CD19^+^ cells (60% *vs* 17%, *P* = 0.02), naïve B cells (54% *vs* 15%, *P* = 0.01), or memory B cells (50% *vs* 16%, *P* = 0.03) 6 months after rituximab. The median relapse free survival time was shorter in patients who failed to achieve complete depletion of CD19^+^ cells (19 *vs* 38 months, *P* = 0.02), naïve B cells (16 *vs* 38 months, *P* = 0.01), or memory B cells (19 *vs* 38 months, *P* = 0.03) 6 months after rituximab.

**Conclusion:**

The degree of B cell depletion 6 months after rituximab may predict disease flare and may instruct on the pacing of B cell depletion therapy in IgG4-RD.

Rheumatology key messages

- Predictors of IgG4-RD relapses after B cell depletion with rituximab are lacking
- Relapses occur earlier in patients who fail to deplete B cells 6 months after rituximab
- Measurement of CD19^+^ B cells may instruct on the pacing of rituximab in IgG4-RD

## Introduction

IgG4-related disease (IgG4-RD) is a relapsing–remitting fibro-inflammatory disorder that leads to tissue fibrosis and organ damage if untreated [1]. B cell depletion therapy with rituximab is effective in most patients but does not prevent disease flares, and repeated infusions are often required to maintain remission [2–4].

The optimal strategy to re-treat IgG4-RD patients with rituximab remains unclear because the time to relapse after each cycle is variable and likely depends on disease intrinsic features (e.g. organ involvement) and on concomitant immunosuppressive drugs. In rheumatoid arthritis, cycles are often given on clinical relapse but this approach may be riskier in IgG4-RD because every other flare may cause life- or organ-threatening complications [5]. Alternatively, pre-emptive treatment with rituximab based on B cell reconstitution or at fixed intervals may be adopted in analogy to other immune mediated disorders. Yet, biomarkers that could guide this decision process in IgG4-RD have not been identified and unnecessary exposure of frail patients to long-term immunosuppression remains an important concern.

In the present study we assessed B cells as potential biomarkers to predict IgG4-RD relapse after rituximab induction of remission. A number of B cell subsets have been, in fact, linked to IgG4-RD activity and implicated in the pathogenesis of IgG4-RD. These cells include naïve and memory B cells, circulating plasmablasts, plasma cells and, more recently, ‘double negative’ B cells [6–9]. B cells can, indeed, contribute to IgG4-RD through different mechanisms such as antigen presentation to pathogenic T lymphocytes, production of autoantibodies and orchestration of tissue fibrosis [10, 11]. Measuring specific B cell subsets to instruct on the optimal timing of rituximab re-administration represents, therefore, a reasonable approach to address a relevant unmet need in the management of patients with IgG4-RD.

## Methods

### Patients

The present retrospective observational study included patients referred to the IgG4-RD Clinic of San Raffaele Scientific Institute (Milan, Italy) between 2015 and 2019. IgG4-RD was diagnosed according to the ‘Consensus Statement on the Pathology of IgG4-RD’, and fulfilment of the ‘2019 ACR/EULAR Classification Criteria’ was retrospectively confirmed [12]. Patients were required to have active IgG4-RD. All patients were treated with two 1000 mg doses of the rituximab (Mabthera^®^) 15 days apart as induction of remission therapy. Premedication with intravenous methylprednisolone (100 mg) and hydroxyzine (100 mg) was administered prior to rituximab. All patients provided written informed consent for the treatment and analyses performed. This research was part of the ‘Pan-immuno’ research protocol, approved by the IRCCS Ospedale San Raffaele Institutional Review Board (reference number: 22/INT/2018) and conforming to the Declaration of Helsinki.

### Disease activity

Disease activity was assessed by means of the IgG4-Responder Index (IgG4-RD RI) as follows: active disease (IgG4-RD RI > 3); partial response (decrease of the IgG4-RD RI > 2 points but still > 3) to treatments; complete response (IgG4-RD RI < 3 on glucocorticoid treatment); disease remission (IgG4-RD RI < 3 off glucocorticoid therapy) [13]. Relapse was defined as the development of radiological findings or biochemical abnormalities consistent with a new or worsening inflammatory process requiring re-treatment or increase in the dose of glucocorticoid therapy.

### Flow cytometry

Total CD19^+^ and CD20^+^ B cells, CD19^+^CD20^−^CD27^+^CD38^++^ plasmablasts, CD19^+^CD20^+^CD27^−^CD38^+^ naïve B cells and CD19^+^CD20^+^CD27^+^CD38^−^ memory B cells from peripheral blood were measured by flow cytometry. B cells were measured at baseline prior to rituximab therapy and at 6 months, a time point when most studies demonstrate their reappearance after initial depletion [14, 15]. Complete depletion of total CD19^+^ B cells or B cell subsets was defined as counts ≤0.0001 × 10^9^ cells/l and repopulation as counts >0.0001 × 10^9^ cells/l.

### Statistical analysis

Statistical analysis was performed using IBM SPSS Statistics for Windows, Version 22.0 (IBM Corp., Armonk, NY, USA). Continuous variables were expressed as median with interquartile range (IQR), and categorical variables were reported as count and percentages. Comparisons of paired median values were performed using Wilcoxon’s test. Kaplan–Meier curves were used to estimate the time to disease relapse. Time to relapse was compared via log rank test. Receiver operating characteristic (ROC) curves were generated for linear predictors generated based on the assumption that ascending rank of B cell subset counts would increase the likelihood of an IgG4-RD diagnosis. Univariate and multivariate Cox regression analyses were performed to identify the prognostic factors of disease relapse. Variables with a *P*-value <0.1 at univariate analysis were included in the multivariate model. For all tests, a *P*-value <0.05 was considered statistically significant.

## Results

### Patient characteristics

Thirty-three patients were included in the study (24 males, 73%). Serum IgG4 levels were elevated in 26 patients (79%) and multiorgan involvement was present in 25 patients (76%). Eighteen patients (54%) had a previous IgG4-RD flare successfully treated with glucocorticoids but showed active disease at the time of rituximab infusion. All patients were naïve to rituximab and had detectable CD19^+^ B cells at baseline. There was no difference in CD19^+^ B cells, circulating plasmablasts, naïve and memory B cells between patients with single or multi organ involvement at baseline. All patients were on glucocorticoids (median prednisone dose 5 mg [IQR 0–14 mg]) but not on immunosuppressive agents at the time of rituximab infusion. Baseline characteristics of the patients’ cohort are described in Supplementary Table 1, available at *Rheumatology* online.

### Treatment outcomes

Six months after rituximab, disease response was observed in all patients. Four patients (12%) achieved partial response, 11 patients (33%) achieved complete response, and 18 patients (54%) reached disease remission. All patients in disease remission were able to withdraw corticosteroid therapy within 4 months after rituximab infusion. Fifteen patients were still on low dose oral prednisone (median 5 mg; range 3–5 mg). The median IgG4-RD RI declined from 9 (IQR 6–9) to 1 (IQR 0–2). At the longest available follow-up, 23 relapses (70%) were observed. The median time to relapse was 16 months (IQR 9–24 months). In particular, eight patients (35%) relapsed within 12 months and 18 patients (78%) relapsed within 24 months. Adverse events were recorded in nine patients (30%), including three rituximab-related infusion reactions, two cases of infectious cholangitis, two cases of pneumonia requiring oral antibiotics. One patient developed pneumonia requiring intravenous antibiotic. No deaths were recorded during the follow-up.

### Predictors of relapse at baseline

**Figure 1.**
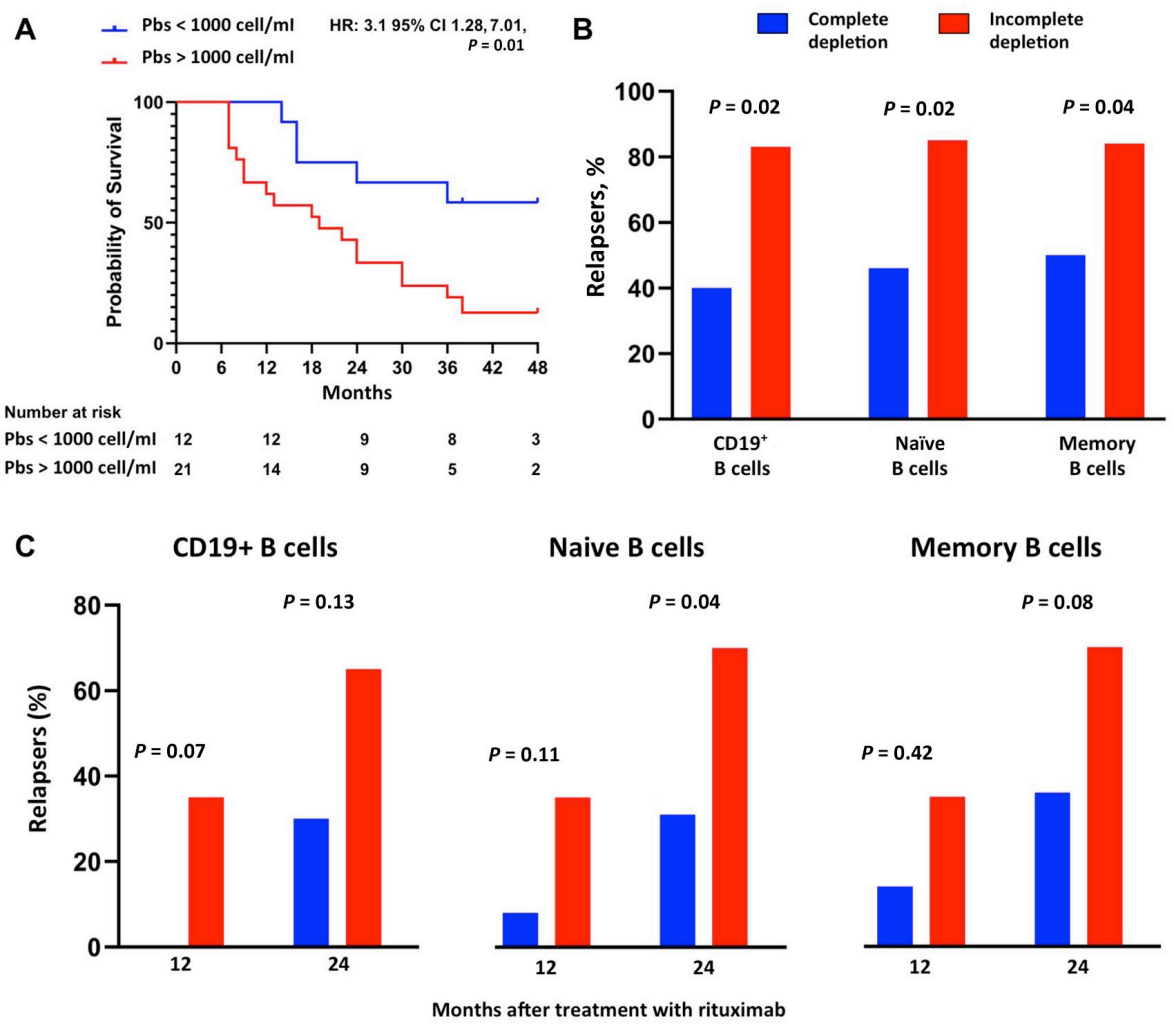
B cell depletion and rate of IgG4-related disease flare. (**A**) Kaplan–Meier survival curve showing the risk of IgG4-RD relapse based on basal levels of circulating plasmablasts. (**B**, **C**) Histograms showing the percentage of IgG4-RD patients relapsing at the longest available follow-up (**B**) or at 12 and 24 months (**C**) after rituximab based on complete or incomplete depletion of total CD19^+^ cells, naïve and memory B cells 6 months after treatment. HR: hazard ratio; Pbs: plasmablast

Baseline plasmablast count was significantly higher in patients experiencing disease relapse (median 2880 cells/ml; IQR 1230–5650 cell/μl) compared with non-relapsers (median 710 cells/ml; IQR 365–1270 cell/ml) (*P* < 0.01). There was no difference in baseline total CD19^+^ cells, naïve B cells and memory B cells between the two groups either in absolute numbers or as a percentage of CD19^+^ B cells (*P* > 0.05 for all comparisons). A plasmablasts count of 1030 cell/ml had a sensitivity of 78% and a specificity of 70% in predicting disease relapse (area under the ROC curve [AUC]=0.815; 95% CI 0.67, 0.96; *P* < 0.01). A plasmablast count higher than 1000 cell/ml represented the only factor associated with disease relapse at the multivariable analysis (odds ratio = 2.72; 95% CI 1.02, 7.45; *P* = 0.04) (Fig. 1A and Supplementary Table 2, available at *Rheumatology* online).

### Predictors of relapse post-rituximab therapy

**Figure 2.**
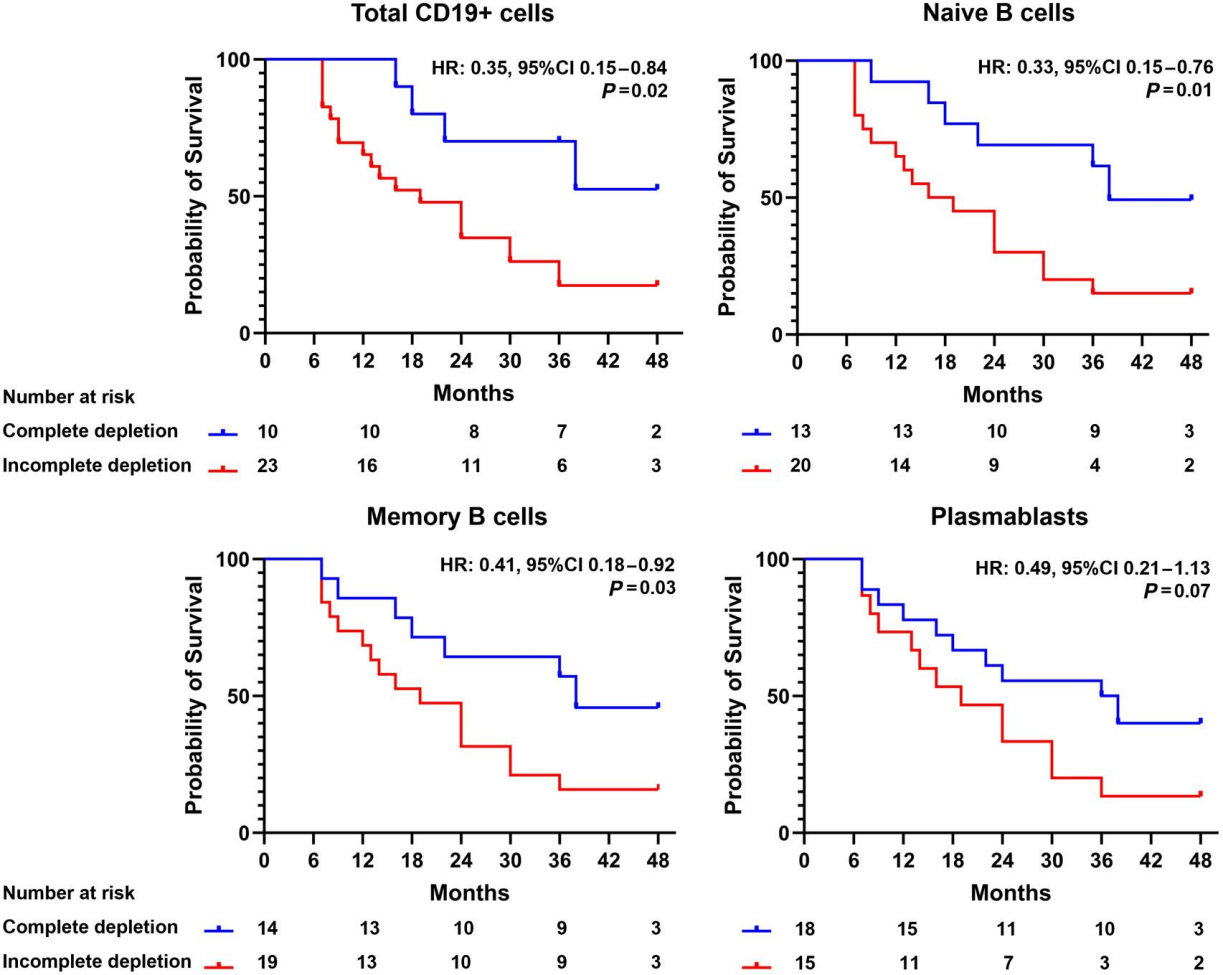
B cell depletion and timing of IgG4-related disease flare. Kaplan–Meier survival curves showing IgG4-RD relapses over time in patients treated with rituximab based on the complete or incomplete depletion of total CD19^+^ cells, naïve and memory B cells 6 months after treatment. HR: hazard ratio

Six months post-rituximab, complete depletion of CD19^+^ B cells was achieved in 10 patients (30%). Plasmablasts, naïve and memory B cell depletion was achieved in 55%, 39% and 42% of cases, respectively. At the longest available follow-up (median 37 months, range 26–49), relapse rate was significantly higher in patients who failed to achieve complete depletion of CD19^+^ cells (83% *vs* 40%, *P* = 0.02), naïve B cells (85% *vs* 46%, *P* = 0.02) or memory B cells (84% *vs* 50%, *P* = 0.04) (Fig. 1B). In particular, among patients with incomplete CD19^+^ B cell depletion at 6 months, eight (35%) relapsed within 12 months, and 15 (65%) within 24 months; among patients with incomplete naïve B cell depletion at 6 months, seven patients (35%) relapsed at 12 months and 14 (70%) at 24 months; among patients with incomplete memory B cell depletion at 6 months, six patients (32%) relapsed at 12 months and 13 (68%) at 24 months (Fig. 1C). The median relapse free survival time was significantly shorter in patients who failed to achieve complete CD19^+^ depletion (19 *vs* 38 months, *P* = 0.02), naïve B cell depletion (17 *vs* 38 months, *P* = 0.01) or memory B cell depletion (19 *vs* 38 months, *P* = 0.03) (Fig. 2). Relapse rate as well as median relapse free survival time did not differ between patients who achieved plasmablast depletion and those who did not. There was no difference in the rate of adverse events according to the B cell depletion status.

## Discussion

IgG4-related disease is a relapsing condition that promptly responds to B cell depletion therapy with rituximab. Repeated infusions of rituximab are necessary in most patients to maintain remission but this comes at the risk of side effects related to prolonged immunosuppression [3]. To avoid overtreatment, a biomarker that predicts response and indicates imminent relapse with sufficient warning to allow for intervention would be of value.

In this study we demonstrate that the degree of CD19^+^ B cell, naïve and memory B cell depletion after rituximab treatment predicts duration of response and patterns of IgG4-RD flare. In particular we show that patients with persistently measurable counts of CD19^+^ B cells, naïve or memory B cells 6 months after rituximab relapse on average within 19 months. These findings have a number of practical implications for the management of patients with IgG4-RD.

First, our work suggests that patients with detectable CD19^+^ B cells 6 months after rituximab may be suited for pre-emptive retreatment (e.g. within 12 months from the previous infusion) due to a 35% relapse rate observed at 1 year and a 65% relapse rate at 2 years. Conversely, those with complete B cell depletion should not be retreated at fixed intervals but might rather benefit from a close watch, waiting to be promptly infused when intercepting incipient flares. Whether this approach could decrease the incidence of rituximab-related side effects remains to be demonstrated in long-term prospective comparative studies.

Second, our work indicates that a lower incidence of flares after rituximab is associated with a more profound depletion of peripheral B cells in analogy to what is already reported, for instance, in lupus nephritis [16]. As persistence of circulating B cells after rituximab is generally attributed to inefficient removal of tissue-resident B-lymphocytes, a deeper B cell depletion such as that obtained by targeting CD19^+^ cells would appear reasonable to improve IgG4-RD outcomes. Indeed, anti-CD19 therapy with inebilizumab is currently being tested in a clinical trial (clinicaltrials.gov: NCT04540497) and obinutuzumab—a humanized anti-CD20 monoclonal antibody with higher B cell depleting activity compared with rituximab—has been proven effective in IgG4-RD [17, 18].

Third, while identification of naïve and memory B cells requires sophisticated cytometry panels and is not standardized across laboratories, measurement of CD19^+^ B cells is easily reproducible and widely used in haematological and immunological settings, representing a cheap and readily available biomarker for predicting disease outcomes also in IgG4-RD.

Finally, as similar studies were previously conducted in patients with other immune-mediated disorders, our results seem to suggest that perturbations of the B cell compartment in IgG4-RD are more similar to those observed in systemic lupus erythematosus (SLE) than those observed in ANCA-associated vasculitis [19, 20]. In ANCA-associated vasculitis, in fact, depletion rather than persistence of naïve B cells 6 months after rituximab predicts early relapse [20]. Although we currently ignore the reasons for these discrepancies, they likely reflect different alterations of B cell subsets among diverse immune-mediated disorders [20].

Interestingly, the level of circulating plasmablasts—the subset of circulating B cells that has been most inherently associated to IgG4-RD activity—correlated with disease relapse when measured at baseline but was not shown to predict relapse when measured after rituximab. This observation is likely due to the fact that plasmablasts do not express CD20—the target of rituximab—and might thus be less directly affected by the treatment showing different depletion kinetics compared with their CD20^+^ precursors.

Our results show significant points of strength but also have some limitations. First, this is the first study, to our knowledge, that correlates the risk of IgG4-RD relapse after rituximab with cellular biomarkers of IgG4-RD. In addition, the present work has been carried out on one of the largest single-centre cohorts of patients with IgG4-RD, an aspect that ensured uniform inclusion criteria, treatment and interpretation of flow-cytometry data. Furthermore, the relapse rate that we observed in our patient population corresponds to that reported in other IgG4-RD cohorts reinforcing the reliability of study outputs [2, 4]. On the other hand, we recognize the retrospective design of the study that prevents definitive conclusions. We also did not investigate additional B cell subsets within the naïve and memory compartments such as double negative B cells, but these cells were first described after the completion of this study. Moreover, our cohort mainly included patients with a pancreato-biliary phenotype, and thus replication of our findings in patients with predominant head and neck and retroperitoneal involvement is warranted. Finally, our study does not instruct about the role of B cell subsets in predicting relapse after multiple rituximab infusions. In this sense a larger study population with a longer follow-up might have provided more robust results.

In conclusion, our study offers new insights into the clinical use of B cell depletion in IgG4-RD with regard to the optimal timing of rituximab re-administration. The practical implications of our experience have the potential to reduce overtreatment and warrant further investigation in larger cohorts to instruct the design of future clinical trials.

## Supplementary Material

keae248_Supplementary_Data

## Data Availability

Data are available upon reasonable request to the corresponding author.
