# Supplementary material for: B cell depletion after treatment with rituximab predicts relapse of IgG4-related disease
Source: Rheumatology (Oxford). 2024 May 23;64(4):2290–4. doi: 10.1093/rheumatology/keae248 (PMC11962880; doi:10.1093/rheumatology/keae248)
Supplement: keae248_Supplementary_Data [file keae248_supplementary_data.zip › keae248_Supplementary_Data/rhe-23-2696-File005.docx]

|  | IgG4-RD patients  (n = 33) |
| --- | --- |
| Age, median (years) | 62 (55-72) |
| Male, n° (%) | 24 (73%) |
| IgG4-RD RI (0-3) | 9 (6-9) |
| Elevated serum IgG4, n (%)  Multiorgan involvement | 24 (73%)  25 (76%) |
| CD19^+^ B cells (cells/mL) | 194000 (110000-306000) |
| CD20^+^ B cells (cells/mL) | 164500 (103000-221700) |
| Naive B cells (cells/mL) | 10250 (6360-25200) |
| Memory B cells (cells/mL) | 24150 (10090-58275) |
| Plasmablasts (cells/mL) | 2010 (710-4660) |
| Glucocorticoids dose (mg) | 5 (0-14) |
| **Organ involvement, n°(%)** |  |
| Pancreas | 21 (64%) |
| Aorta and retroperitoneum | 8 (24%) |
| Lymph nodes | 7 (21%) |
| Biliary tree | 7 (21%) |
| Salivary glands | 4 (12%) |
| Lacrimal glands | 4 (12%) |
| Lung | 2 (6%) |
| Orbit | 2 (6%) |
| Nasal Sinuses | 2 (6%) |
| Meninges | 2 (6%) |
| Kidney | 1 (3%) |
|  |  |

**Supplementary Table 1.** Clinical and laboratory features of the patients' cohort at baseline. Data are reported as median and IQR or counts and frequencies. Abbreviations. IgG4-RD RI: IgG4-related disease Responder Index
