# Supplementary material for: B cell depletion after treatment with rituximab predicts relapse of IgG4-related disease
Source: Rheumatology (Oxford). 2024 May 23;64(4):2290–4. doi: 10.1093/rheumatology/keae248 (PMC11962880; doi:10.1093/rheumatology/keae248)
Supplement: keae248_Supplementary_Data [file keae248_supplementary_data.zip › keae248_Supplementary_Data/rhe-23-2696-File006.docx]

|  | Univariate analysis | P value | Multivariable analysis | P value |
| --- | --- | --- | --- | --- |
| Age | 1.01 (0.98-1.04) | 0.49 |  |  |
| Gender | 1.98 (0.83-4.7) | 0.12 |  |  |
| N°organs involved | 1.68 (0.89-3.19) | **0.09** | 1.56 (0.81-3.06) | 0.19 |
| IgG4-RD RI | 1.13 (0.96-1.32) | 0.13 |  |  |
| CD19 | 1 (0.99-1.01) | 0.81 |  |  |
| Plasmablast > 1000 cell/mL | 3.28 (1.2-8.91) | **0.02** | 2.72 (1.02-7.45) | **0.04** |
| Naïve B cells | 1 (0.99-1.01) | 0.44 |  |  |
| Memory B cells | 1 (0.99-1.01) | 0.42 |  |  |
| Elevated serum IgG4 | 1.84 (0.62-5.43) | 0.27 |  |  |

**Supplementary Table 2.** **Univariate and multivariate analysis for evaluating baseline risk factors for relapse.** Variables with a P < 0.1 at univariate analysis were included in the multivariate model**.**
